# Heavy metal blood concentrations in smelters and chimney sweepers in Norway

**DOI:** 10.1093/annweh/wxaf089

**Published:** 2026-01-21

**Authors:** Krister Aune Teigen, Anje Christina Höper, Sandra Huber, Marit Nøst Hegseth

**Affiliations:** Department of Occupational and Environmental Medicine, University Hospital of North Norway, Pb 16, 9038 Tromsø, Norway; Department of Community Medicine, Faculty of Health Sciences, UiT the Arctic University of Norway, 9037 Tromsø, Norway; Department of Occupational and Environmental Medicine, University Hospital of North Norway, Pb 16, 9038 Tromsø, Norway; Department of Community Medicine, Faculty of Health Sciences, UiT the Arctic University of Norway, 9037 Tromsø, Norway; Department of Laboratory Medicine, University Hospital of North Norway, Pb 100, 9038 Tromsø, Norway; Department of Occupational and Environmental Medicine, University Hospital of North Norway, Pb 16, 9038 Tromsø, Norway; Department of Community Medicine, Faculty of Health Sciences, UiT the Arctic University of Norway, 9037 Tromsø, Norway

**Keywords:** heavy metals, biological monitoring, particulate matter, occupational exposure

## Abstract

**Background:**

Smelter workers and chimney sweepers may be exposed to heavy metals via particulate matter through airways and skin uptake. We hypothesized that these workers have higher blood concentrations of arsenic, cadmium, chromium, lead, and mercury compared to an office-worker group. Furthermore, we hypothesized that their concentrations increased cross-week despite the use of properly fitted respiratory protective equipment (RPE). Our aim was to uncover workers’ general heavy metal concentrations and cross-week changes to provide further insight into industrial occupational exposure.

**Methods:**

We conducted a cross-sectional study with 2 repeated measures. We quantified whole blood arsenic, cadmium, chromium, lead, and mercury 4 d apart. Participants answered a standardized questionnaire and log scheme on work exposure. Samples were analyzed using inductively coupled plasma mass spectrometry, and we used Wilcoxon's signed-rank test and multiple linear regression to assess heavy metal differences across the 4-d workweek and occupational groups.

**Results:**

Out of 226 participants (99 smelter workers, 80 chimney sweepers, and 47 office workers), 185 participants provided blood samples. The final dataset included 151 participants. Overall, heavy metal concentrations were below Mayo Clinic's cutoff concentrations. Multiple linear regression analysis with confounders did not show higher concentrations of heavy metals in the exposed occupations compared to office workers. Instead, we found statistically significant lower chromium in smelter workers. Sensitivity power analysis showed that our nonsignificant findings in the other metals were underpowered. Wilcoxon's signed rank test did not show cross-week increase.

**Conclusions:**

In our population of chimney sweepers and smelter workers with high percentage use of RPE, occupational exposure to heavy metals does not seem to increase workers blood heavy metals; lifestyle factors seem more important. Further studies with higher number of participants are warranted to ensure our findings.

What's Important About This Paper This study found that the concentrations of metals in the blood of smelter workers and chimney sweeps were not higher than among a sample of office workers. This suggests that lifestyle factors are more important for total exposure than occupational exposures among this sample with high use of respiratory protection. This finding is surprising based on known exposure in these industries, but there has been a lack of information about metal exposures among these workers.

## Introduction

Heavy metals, while essential for human civilization, are also among the oldest known toxicants. Heavy metals may be present as liquid, gas, or bound to particulate matter. In the occupational setting inhalation is the primary route of uptake of heavy metals, but there are also other possible routes, such as skin absorption through exposure to nanoparticles (PM0.1) (Baroli et al. 2007; Larese et al. 2009; Larese Filon et al. 2011, 2013; Liang et al. 2013; Brouwer et al. 2016; Wahlqvist et al. 2020; Afzal et al. 2022). Due to PM0.1's small mass and large surface area, they behave differently than larger particles, with the potential of carrying higher concentrations of chemicals and metals (Brown et al. 2000; Li et al. 2003; Monteiller et al. 2007; Larese Filon et al. 2016). The small size even enables them to penetrate healthy skin (Baroli et al. 2007; Crosera et al. 2009; Larese et al. 2009; Larese Filon et al. 2011, 2013; Liang et al. 2013; Wahlqvist et al. 2020; Zoabi et al. 2021). Workers in industries with particulate matter exposure, such as smelters and chimney sweepers, may therefore be exposed to heavy metals through their work despite their mandatory use of respiratory protective equipment (RPE).

### Ferro-silica smelting

Historically, smelter production is associated with exposure to particulate matter. Ferrosilicon (FeSi), an alloy used to stabilize cement, is produced using quartz, coal, iron ore, coke, and biocarbon (Eric 2014; Kero et al. 2017). Quartz, a crystalline mineral composed of silicon dioxide (SiO_2_), is reduced with carbon in the presence of iron at almost 2,000 °C to produce the alloy. The process generates particulate matter and gases that are mainly made up of silicon monoxide (SiO), amorphous silica (SiO_2_), polycyclic aromatic hydrocarbons (PAHs), and heavy metals. The raw materials may be contaminated with heavy metals, such as zink (Zn), lead (Pb), cadmium (Cd), and manganese (Mn), which may escape as vapors or are bound to particulate matter (Eric 2014; Kero et al. 2017). Airborne emissions may contain chromium, arsenic, mercury, lead, and cadmium. As far as we know, no studies have investigated the heavy metal content in the particulate matter present within the furnace hall, only inside the furnace itself (Næss et al. 2013, 2014; Eric 2014; Kero et al. 2017; Jorgensen et al. 2020).

Previous studies on smelter workers have found increased risk of heart-, lung-, and neurological disease, different cancers, and kidney damage (Steenland et al. 1992; Gibbs et al. 2014). Over the last decade, there has been a focus on preventive measures, such as fume mitigation and optimized use of RPE in the Norwegian smelting industry. A previously published work conducted in the same smelter plant of the present study showed that a knowledge-based intervention increased the workers’ use of RPE substantially (Robertsen et al. 2020) even though RPE use was already mandatory.

Smelter plants house a diverse workforce with varying levels of exposure to potential hazards depending on their roles and work locations. These roles typically include mechanics, electricians, process operators, oven workers, and those involved in transportation and storage. Even within these categories, exposure can differ significantly based on the specific tasks performed, such as welding, electrical work, or mechanical maintenance.

### Chimney sweeping

Chimney sweepers are mainly exposed to particulate matter through soot. Soot is a black particulate matter that is formed as a byproduct of combustion or pyrolysis of organic materials such as coal, wood, fuel oil, waste oil, paper, plastics, and household refuse (IARC Working Group on the Evaluation of Carcinogenic Risks to Humans 2012). The soot consists of up to 60% carbon, but may contain metals such as arsenic, nickel, lead, chromium, and cadmium (Andersson 1987; Berglund 2018). Depending on the source, other compounds such as hydrogen chloride (plastic/rubber) and PAHs may be present in the soot (IARC Working Group on the Evaluation of Carcinogenic Risks to Humans 2012). These compounds will accumulate in chimneys over time, and sweeping will release the compounds to the air around the sweeper. Chimney sweepers primarily employ 2 methods: traditional rope and brush systems (using metal or synthetic brushes) and power sweeping techniques involving rotating brushes. Both methods produce particulate matter, such as nanoparticles and others, to a great extent (Moazami et al. 2023). The larger particles move downwards through the chimney, while smaller particles ascend. After sweeping, sweepers empty the soot from the bottom of the chimney.

Historically, chimney sweepers had a high risk of scrotal cancer (Benmoussa et al. 2019). Newer studies found increased health risks of esophageal and gastric cancer, bowel cancer, lung cancer, asthma, chronic obstructive pulmonary disease, emphysema, ischemic heart disease, and more (Jansson et al. 2012). The International Agency for Research on Cancer (IARC) has classified soot, as a group 1 carcinogenic to humans (IARC Working Group on the Evaluation of Carcinogenic Risks to Humans 2012). Similar to the smelter industry, the use of RPE has increased substantially over the years, protecting against inhalation of particulate matter (Alhamdow et al. 2017).

Most studies conducted on heavy metals in blood have investigated nonoccupational exposure in general populations. There are generally very few published studies on exposure in or around occupational settings. We have only found one study on ferro-silica plants sampling external soil (Mishra et al. 2007), one measuring heavy metals in samples from the vicinity of a ferro-manganese factory (Soltan et al. 2005), and one measuring blood concentrations of heavy metals in a population living around a ferro-manganese production site (Markiv et al. 2022). In terms of workers’ exposure, a recent Swedish study correlated cobalt in biological samples with cobalt in air measurements in a Swedish cobalt alloy plant (Wahlqvist et al. 2020), while a Norwegian study examined cross-shift/cross-week measurements of blood concentration of chromium in steel welders (Stridsklev et al. 2004). These studies indicate that workers in the metal alloy industries, especially welders, are at risk of exposure to heavy metals from dust and fumes in their work atmosphere. Nevertheless, knowledge on cross-week exposure measurements of heavy metals in an occupational setting is very scarce and needs further investigation.

### Heavy metals and biomonitoring

Biomonitoring of heavy metals is complex. Sources of heavy metal exposures from home or work, through vapors, gases, particulate matter, dietary intake, water, supplements, or other factors cannot easily be distinguished through conventional blood sampling without isotope-related analysis or methods of source tracking (Fisher and Gupta 2025). All heavy metals have different half-lives within the organism, and some types of heavy metals accumulate in targeted organs such as bone (lead), kidney (cadmium and mercury), and liver (cadmium) where they may have half-lives of 10 to 30 yr, and the heavy metals may leak from bones to the bloodstream (Casarett 2013). There are differences in heavy metal toxicity based on their ionic state and form. This means whether they are in their organic, inorganic, or elemental form. Differentiating the forms in blood requires speciation, which is expensive. Examples of metal toxicity differences are elemental mercury (Hg^0^) that is considered toxic through vapors in their organic, but not in their inorganic form (Casarett 2013). Also, arsenic and inorganic arsenic compounds are considered more toxic than other types of arsenic, and are classified as group 1 carcinogens by IARC (International Agency for Cancer Research 2012). Arsenic’s half-life in blood varies from 3 to 6 h up to 20 h in blood (Martinez-Morata et al. 2023). Chromium is an essential metal in its trivalent ionic state (Cr^3+^), while hexavalent Chromium (Cr^6+^) is considered the toxic form of the metal. Chromium's half-life varies from 15 to 41 h (Agency for Toxic Substances and Disease Registry (ATSDR) 2012a). Cadmium has a half-life in blood for 3 to 4 mo (Agency for Toxic Substances and Disease Registry (ATSDR) 2012b). Inorganic lead is considerably more toxic than the organic state of lead (Casarett 2013; Balali-Mood et al. 2021; Martinez-Morata et al. 2023) and is classified as a group 1 carcinogenic by IARC (International Agency for Cancer Research 2023), and it has a half-life of 1 to 3 mo (Casarett 2013). These circumstances further complicate the biomonitoring of heavy metals.

### Aim

Our study sought to gain knowledge on general and cross-week heavy metal concentrations in an occupational setting. The aim was to measure smelter workers and chimney sweepers and whole blood total concentrations of arsenic, cadmium, chromium, lead, and mercury, comparing them to a PM-unexposed office-worker group. The primary hypothesis was that employees in the industrial occupations had higher blood concentrations than the unexposed office-worker group and that their heavy metal blood concentrations increased during a work week of 4 d, despite the use of properly fitted RPE.

## Materials and methods

### Study participants

We invited 99 smelter workers and 80 chimney sweepers to participate. We recruited workers who have had 2 d off and were actively employed in their respective roles before we collected blood samples. Participants were recruited through contact with leaders at the respective locations. For comparison, we recruited a group of 44 office workers with no expected workplace exposure to particulate matter from the University Hospital of Northern Norway (UNN) and UiT, The Arctic University of Norway. We strived to select the group to match the age and gender distribution of the industrial workers as closely as possible.

We collected information about their potential exposure to particulate matter through questionnaires and work logs. We also asked participants to avoid activities that could generate particulate matter (eg bonfires, barbequing) for 3 d prior to sample collection.

### RPE fit test

Before collecting blood samples, we performed standardized RPE fit tests on all industrial workers using the TSI PortaCount 8038, in accordance with HSE standard (Foereland et al. 2019). The aim of the fit test was to ensure every worker had a mask that fitted them properly and to minimize inhalation exposure during the cross-week sampling. We instructed participants to wear their RPEs as much as possible and to record their usage in the provided work logs.

### Blood sampling

We sampled blood from participants before they entered their first work shift and immediately after their fourth shift—after 4 workdays. This sampling schedule accommodated the work patterns of both smelter workers and chimney sweepers and was their longest period of consecutive workdays, equivalent to a cross-week sampling period to minimize potential skin contamination, we asked workers to shower before each blood draw. This helped ensure that any detected heavy metals truly reflected internal levels rather than surface particles.

We collected blood samples with a BD Vacutainer Safety-Lok blood collection set (ref: 367282) with BD Vacutainer One Use Holder (ref: 364815). Blood was collected into one 6 mL 13 × 100 mm BD Vacutainer Trace element K2 EDTA 10.8 mg (ref: 368381). We inverted Vacutainers 7 times immediately after collection and aliquoted to 2 sterile Sarstedt CryoPure Tube 1.8 mL white (ref: 72.379) using sterile disposable plastic Pasteur pipettes. Then labeling the Cryopure tubes with their respective unique ID-codes and placing them in Thermo Scientific boxes in a refrigerator between 2 to 6 °C for 0 to 4 d before freezing at −33 °C, awaiting analysis. Studies have proven stability of metals in blood of up to 36 mo at refrigeration and freezing temperatures (Tevis et al. 2018; Tanvir et al. 2021). The different latency from sampling to freezing was due to logistic and transport of the samples from location to the laboratory.

Participants who exceeded the reference levels established by the Mayo Clinic (Mayo Clinic Laboratories) were invited for a follow-up 12 to 18 mo after the initial sampling.

### Blood analysis

Blood samples were analyzed at the Environmental Pollutant Laboratory, Department of Laboratory Medicine, at the UNN, Tromsø, Norway, according to a previously published method (Huber et al. 2024). Blood samples were thawed and aliquots of 200 µL were diluted 1:20 on a liquid handler station prior instrumental analysis on Nexion 300D inductively coupled-plasma mass-spectrometer (Perkin Elmer, Waltham, MA, USA). The diluted samples were analyzed in kinetic energy discrimination mode with helium as reaction gas and with low (4.8 mL) and high (5.7 mL) helium flow and 1600W RF generator power. Rhodium and rhenium (Inorganic Ventures, Christiansburg, VA, USA) as internal standards. We applied a 4-point matrix-matched calibration curve with linear regression through zero, together with a blank subtraction after performance of the internal standard correction for quantification.

Four procedure blank samples and 2 sets of ClinChek and Seronorm control material level 1, level 2, and level 3 were used for quality assurance and quality control with every batch of samples. Procedure blank samples were used for controlling the overall background contribution and for calculation of the method detection limits (MDLs). MDLs were calculated as 3 times standard deviation of the concentrations of the procedure blank samples. Diluent blanks for control of the background and instrumental carry over was included. The average coefficients of variation (CVs) for all the control samples (ClinChek level 1 to 3 and Seronorm level 1 to 3 for whole blood) were: 8% for As, 13% for Cd, 11% for Cr, 9% for Hg, and 5% for Pb. The laboratory participates successfully in the international quality control program Quebec Multielement External Quality Assessment Scheme organized by the Centre de Toxicologie du Quebec, Quebec, Canada. This program covers elemental analysis in human whole blood, serum, and urine.

### Questionnaire

We collected information about participants’ health and potential exposures through an expanded version of Nordic Occupational Skin Questionnaire-2002 (NOSQ-2002) (Susitaival et al. 2003). We expanded with questions covering previous diseases, dietary intake of foods known to contain heavy metals, occupational and non-occupational exposure to particulate matter, tobacco use, time spent in the current occupation, medical history, and more. We distributed paper copies of the questionnaire to participants and collected them on the fourth day of the study. Answered questionnaires were machine-scanned and results were collected in a SPSS file.

### Log scheme

We provided smelter workers and chimney sweepers with tailored standardized work logs. They were asked to document details about their daily tasks, such as welding, number of chimneys swept throughout a workday, the total time spent in areas with particulate matter exposure working inside the furnace, and their use of RPE. They reported their daily percentage of time they wore their RPE in exposed areas. This detailed logging provided us with the possibility to exclude workers who were not or minimally exposed during the study week and possibly associate particulate matter exposure to blood results. Furthermore, it provided us with participants’ adherence to protective measures.

### Statistical analysis

We conducted all statistical analyses using Stata/MP 16.1 by Stata Corp LLS. We identified potential confounders and mediators using Dagitty 3.0 and included these factors in the population characteristics presented in Table 1.

**Table 1:** Characteristics of study population.

Occupation	Smelter worker		Chimney sweeper		Unexposed		Total		Stat.
n	42		65		44		151		test
	*n*	%	*n*	%	*n*	%	*n*	%	χ^2^
Gender									
Man	41	97.6%	56	86.2%	39	88.6%	136	90.1%	0.02
Woman	1	2.4%	9	13.8%	5	11.4%	15	9.9%	
Education									
College/university > 2 yr	1	2.4%	0	0.0%	22	50.0%	23	15.2%	<0.001
College/university < 2 yr	2	4.8%	12	18.5%	14	31.8%	28	18.5%	
Highschool	37	88.1%	54	83.1%	8	18.2%	99	65.6%	
Elementary school	2	4.8%	5	7.7%	0	0.0%	7	4.6%	
Tobacco smoke									
Daily	5	11.9%	3	4.6%	3	6.8%	11	7.3%	<0.001
Occationally	7	16.7%	7	10.8%	5	11.4%	19	12.6%	
Previously	14	33.3%	14	21.5%	4	9.1%	32	21.2%	
Never	16	38.1%	41	63.1%	32	72.7%	89	58.9%	
Snus									
Daily	14	33.3%	27	41.5%	6	13.6%	47	31.1%	<0.001
Occationally	2	4.8%	5	7.7%	3	6.8%	10	6.6%	
Previously	2	4.8%	7	10.8%	8	18.2%	17	11.3%	
Never	24	57.1%	26	40.0%	27	61.4%	77	51.0%	
Seafood intake last week (yes)	2	4.8%	10	15.4%	8	18.2%	20	13.2%	0.022
Gun shooting last week (yes)	0	0.0%	2	3.1%	0	0.0%	2	1.3%	
Welding (yes)	10	23.8%	1	1.5%	0	0%	11		<0.001
-Welding hours last week	6.4	(1–33)	1	(1, 1)	0	0	5.9	(1–33)	

	Mean	(Range)	Mean	(Range)	Mean	(Range)	Mean	(Range)	*P*-value^b^
BMI (kg/m^2^)	29.1	(19.7–55.3)	27.2	(19.6–46.3)	26.3	(20.5–40.4)	27.5	(19.6–55.3)	<0.01
Age (yr)	36.0	(18–67)	37.3	(19–60)	43.7	(26–65)	38.8	(18–67)	<0.01
Years in occupation	9.7	(0.2–37)	10.5	(0.2–35)	9.7	(1–30)	10	(0.2–37)	0.839
Work hours per week (2 missing)	34.9	(20–40)	37.5	(30–40)	38.9	(18.5–70)	37.2	(18.5–70)	<0.001
Particulate matter exposure at work (h/d)	4.9	(0.5–9.25)	2.4	(0–5)	0.0	0	2.4	(0–9.25)	0.610
Particulate matter exposure at home (h/d)^a^	9.9	(0–144)	9.0	(0–79)	7.4	(0–42)	8.8	(0–144)	<0.001
Blood selenium (µg/L) (1 missing)	103.2	(71.49–145.52)	98.7	(60.84–137.31)	99.7	(66.34–147.64)	100.68	(60.84–147.64)	0.261

^a^Bonfire outside, barbequeing, candle burnin indoor, fireplace burning.

^b^ANOVA, Tukey's HSD for comparing groups.

To ensure a complete dataset, we applied the following exclusion criteria:

1. *Missing essential questionnaire data*: We excluded participants with missing data on any of the factors listed in Table 1, except for body mass index (BMI). We excluded 5 chimney sweepers, 2 smelter workers, and 1 office worker. Nine participants lacked height or weight data. In these participants, we interpolated BMI using the available measurement.
2. *Missing blood samples*: This resulted in exclusion of 3 smelter workers, 6 chimney sweepers, and 2 office workers.
3. *Unsatisfactory work location*: We excluded 16 workers who did not work inside the furnace hall daily during the sampling period based on their log scheme.

**Figure 1:**
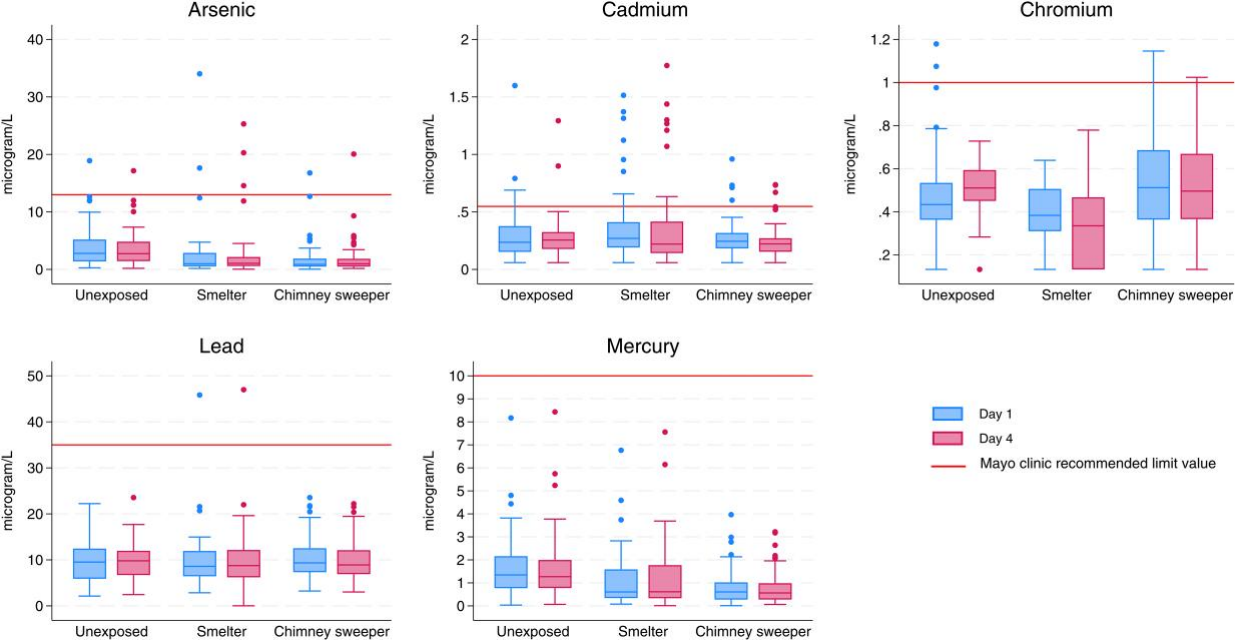
Boxplot of Day 1 (blue) and Day 4 (pink) for each occupational group with limit concentrations given by the Mayo Clinic (Solid line).

We assessed differences in the study population using Chi-square test and ANOVA based on unequal variances (Table 1) with Tukey's HSD for comparing groups. We replaced blood concentrations below the detection limit (LOD) with 1/2LOD. To present general blood concentrations, we used the mean of the 2 sampling days for each participant, with results presented in Fig. 1 and Table 2.

**Table 2:** Mean metal concentrations of Day 1 and 4 combined for each occupational group with their respective distribution (geometric and arithmetic mean, 5th to 95th percentile) together with reference concentrations from Mayo Clinic (^a^), HUNT3 (^b^) and the Norwegian blood donor study (^c^). In general, the geometric mean and median blood concentrations were below the Mayo clinic reference concentrations (Mayo Clinic Laboratories) and the HUNT3 geometric mean in all occupational groups (Syversen et al. 2021).

Overall *n* = 151												
Element	LOD	<LOD	Range	GM	AM	Percentiles (µg/L)				Ref. level^a^	HUNT3^b^	Blood donor^c^
	(µg/L)	%	(µg/L)	(µg/L)	(µg/L)	5%	25%	75%	95%	(µg/L)	GM [5th to 95th]	AM [range]
Arsenic	0.08	1.0%	<0.08–34.02	1.36	2.72	0.23	0.56	3.02	11.90	<13	2.64 (0.61–14.50)	*−*
Cadmium	0.12	10.9%	<0.12–1.77	0.24	0.31	<0.12	0.16	0.34	0.90	<0.47	0.36 (0.11–1.87)	0.39 [<0.11–3.89]
Chromium	0.26	13.6%	<0.26–1.18	0.42	0.47	<0.26	0.34	0.60	0.82	<1	0.58 (0.4–2.26)	*−*
Lead	0.04	0.3%	<0.04–47.00	8.80	10.01	4.27	6.45	12.29	19.46	<35	18.8 (8.9–45.5)	13.8 [<3.3–121.3]
Mercury	0.02	0.7%	<0.02–11.65	0.70	1.19	0.09	0.34	1.56	3.69	<10	2.74 (0.84–9.69)	2.48 [<0.19–25.9]

Smelter workers *n* = 42									
Element	LOD	>LOD	Range	GM	AM	Percentiles (µg/L)			
	(µg/L)	%	(µg/L)	(µg/L)	(µg/L)	5%	25%	75%	95%
Arsenic	0.08	1.2%	<0.08–34.02	1.24	2.93	0.22	0.57	2.73	14.57
Cadmium	0.12	10.7%	<0.12–1.77	0.27	0.39	<0.12	0.16	0.41	1.31
Chromium	0.26	27.4%	<0.26–0.78	0.32	0.36	<0.26	<0.26	0.49	0.64
Lead	0.04	1.2%	<0.04–47.00	8.33	10.20	3.90	6.23	12.12	20.69
Mercury	0.02	1.2%	<0.02–11.65	0.66	1.22	0.08	0.34	1.65	3.74

Chimney Sweepers *n* = 65									
Element	LOD	>LOD	Range	GM	AM	Percentiles (µg/L)			
	(µg/L)	%	(µg/L)	(µg/L)	(µg/L)	5%	25%	75%	95%
Arsenic	0.08	1.5%	<0.08–20.07	0.98	1.78	0.23	0.56	1.70	5.33
Cadmium	0.12	10.0%	<0.12–0.96	0.22	0.26	<0.12	0.19	0.28	0.59
Chromium	0.26	10.8%	<0.26–1.15	0.46	0.53	<0.26	0.38	0.67	0.89
Lead	0.04	0.0%	3.03–23.54	9.28	10.20	4.56	7.23	12.58	19.98
Mercury	0.02	0.8%	<0.02–3.97	0.49	0.79	0.08	0.27	0.09	2.11

Unexposed *n* = 44									
Element	LOD	>LOD	Range	GM	AM	Percentiles (µg/L)			
	(µg/L)	%	(µg/L)	(µg/L)	(µg/L)	5%	25%	75%	95%
Arsenic	0.08	0.0%	0.19–18.90	2.41	3.90	0.32	1.44	4.92	12.00
Cadmium	0.12	12.5%	<0.12–1.60	0.23	0.31	<0.012	0.17	0.35	1.60
Chromium	0.26	4.5%	<0.26–1.18	0.46	0.49	0.28	0.40	0.57	0.79
Lead	0.04	0.0%	2.13–23.54	8.56	9.55	2.86	6.38	12.34	17.11
Mercury	0.02	0.0%	0.03–8.44	1.21	1.75	0.20	0.78	2.06	4.80

^a^Reference level Mayo Clinic.

^b^HUNT3 geometric means.

^c^Norwegian blood donor study (converted from nmol/L and µmol/L).

We performed multiple linear regression to compare mean heavy metal concentrations across the occupations, adjusting for the factors in Table 1. Based on non-normal distribution of arsenic, cadmium, mercury, and lead concentrations, we log-transformed (natural log) all heavy metal results for simplicity. We checked the normality of residuals both statistically and visually, using the Shapiro–Wilk test, kernel density distribution plots, and PP plots, respectively. We assessed heteroscedasticity using Cameron & Trivedi's decomposition of the IM test. We checked for multicollinearity using a variance inflation factor < 10 and checked visually for linearity using locally weighted scatterplot smoothing (Lowess).

Post regression, we conducted a sensitivity power analysis to estimate the minimum required *R*^2^ for the regression model and the minimum effect size of the individual predictors (delta *R*^2^) to be powered at a minimum of 80%. We used GPower (version 3.1) and used the *F*-test, fixed model, *R^2^* deviation from zero for linear multiple regression (Supplementary Materials) together with manual calculation of the delta *R*^2^ for each significant predictor. Minimum *R*^2^ for the whole regression model was calculated to 17.7% in lead and 17.4% in the other metals. Minimum individual delta *R^2^* was calculated to 5.03% (Supplementary Materials). To investigate if we could find a cross-week difference, we used Wilcoxon's signed-rank test where each participant was their own matched pair. Results are visualized the results in Table 2, including *z*-score and *P*-values from the test.

## Results

The total number of participants was 186. Based on exclusion criteria, our final dataset included 151 participants: 42 smelter workers, 65 chimney sweepers, and 44 office workers. Characteristics of the study population are found in Table 1.

Participation rates before exclusion were 63.6% and 95.0% in smelter workers and chimney sweepers, respectively. After exclusion, participation rates were 42.4% and 81.3%, respectively. For the unexposed group, we do not have the number of invitees; thus, this is not reported.

The PM-exposed professions were male-dominated. There were statistically significant differences between the groups in educational level, tobacco smoking, the use of snus, BMI, age, workhours per week, PM exposure at work, and welding conducted last week. There were no differences in years in occupation, but smelter workers had statistically significant fewer work hours per week than the other 2 groups and conducted welding significantly more often. In addition, particulate matter-exposed workers reported using RPE more than 95% of the time in exposed areas throughout the sampling period (data not shown).

### Blood heavy metal concentrations

The investigated heavy metals were detected at concentrations above lower detection limit (LOD) in 86.4% to 100% of all blood samples. Figure 1 shows a boxplot of heavy metals from each sampling day (1 and 4) with limit values from the Mayo Clinic. In Table 2, we have taken the mean of Day 1 and Day 4 for each participant for comparison of general heavy metal concentrations.

### Factors associated with concentrations of heavy metals in the study population

Table 3 shows the regression coefficients for the multiple linear regression analysis on the difference of the heavy metal concentration across the occupational groups.

**Table 3:** Linear regression analysis.

Variable	Arsenic				Cadmium				Chromium				Lead				Mercury			
	Coeff	Std. error	*P*-value	(95% CI)	Coeff	Std. Error	*P*-value	(95% CI)	Coeff	Std. error	*P*-value	(95% CI)	Coeff	Std. error	*P*-value	(95% CI)	Coeff	Std. error	*P*-value	(95% CI)
Occupation																				
Office workers	Ref				Ref				Ref				Ref				Ref			
Smelter workers	0.48	0.36	0.18	(−0.22 to 1.18)	−0.152	0.19	0.416	(−0.52 to 0.22)	**−0**.**45**	0.16	**0**.**007**	**(−0.77 to −0.13)**	0.03	0.19	0.852	(−0.33 to 0.40)	0.53	0.34	0.12	(−0.14 to 1.19)
Chimney sweepers	−0.07	0.27	0.804	(−0.59 to 0.46)	−0.13	0.14	0.417	(−0.39 to 0.16)	−0.04	0.12	0.749	(−0.28 to 0.20)	0.17	0.14	0.213	(−0.10 to 0.45)	−0.03	0.25	0.894	(−0.53 to 0.47)
Gender																				
Male	Ref				Ref				Ref				Ref				Ref			
Female	−0.34	0.27	0.15	(−0.82 to 0.13)	0.09	0.13	0.479	(−0.16 to 0.34)	−0.10	0.1	0.353	(−0.32 to 0.11)	**−0**.**35**	0.12	**0**.**006**	**(−0.60** to **−0.11)**	−0.12	0.22	0.59	(−0.57 to 0.33)
Education																				
College/University > 2 yr	Ref				Ref				Ref				Ref				Ref			
College/University <2 yr	−0.49	0.28	0.08	(−1.04 to 0.06)	0.22	0.15	0.130	(−0.07 to 0.51)	0.04	0.13	0.773	(−0.21 to 0.29)	−0.06	0.15	0.659	(−0.35 to 0.22)	−0.36	0.26	0.175	(−0.88 to 0.16)
High school	−0.33	0.32	0.3	(−0.96 to 0.30)	0.15	0.17	0.361	(−0.18 to 0.48)	0.09	0.15	0.523	(−0.19 to 0.38)	−0.05	0.17	0.770	(−0.38 to 0.28)	−0.26	0.3	0.398	(−0.85 to 0.34)
Elementary school	−0.77	0.46	0.095	(−1.70 to 0.14)	−0.09	0.24	0.712	(−0.57 to 0.39)	0.02	0.21	0.925	(−0.40 to 0.44)	0.006	0.24	0.979	(−0.47 to 0.48)	−0.44	0.44	0.321	(−1.30 to 0.43)
Smoking																				
Never	Ref				Ref				Ref				Ref				Ref			
Previously	−0.25	0.22	0.252	(−0.69 to 0.18)	**0**.**24**	0.11	**0**.**036**	**(0.02–0.47)**	−0.10	**0**.**1**	0.336	(−0.30 to 0.10)	**0**.**23**	**0**.**11**	**0**.**05**	**(0.0004–0.45)**	−0.09	**0**.**21**	0.670	(−0.50 to 0.32)
Sometimes	0.19	0.25	0.46	(−0.31 to 0.69)	**0**.**64**	0.13	**0**.**000**	**(0.38–0.90)**	0.13	**0**.**12**	0.264	(−0.10 to 0.36)	**0**.**29**	**0**.**13**	**0**.**029**	**(0.03–0.56)**	0.18	**0**.**24**	0.466	(−0.30 to 0.65)
Daily	**−0**.**68**	**0**.**30**	**0**.**03**	**(−1.27** to **−0.08)**	**1**.**30**	**0**.**16**	**0**.**000**	**(0.99–1.62)**	−0.18	**0**.**14**	0.195	(−0.45 to 0.09)	**0**.**32**	**0**.**16**	**0**.**041**	**(0.014–0.63)**	−0.05	**0**.**28**	0.873	(−0.61 to 0.52)
Snus																				
Never	Ref								Ref				Ref				Ref			
Previously	0.2	0.25	0.43	(−0.30 to 0.70)	−0.22	0.13	0.092	(−0.49 to −0.04)	−0.04	0.12	0.721	(−0.27 to 0.19)	0.01	0.13	0.932	(−0.25 to 0.27)	0.18	0.24	0.465	(−0.30 to 0.64)
Sometimes	0.19	0.29	0.525	(−0.42 to 0.74)	0.12	0.15	0.428	(−0.18 to 0.43)	−0.01	0.13	0.928	(−0.28 to 0.25)	0.05	0.16	0.761	(−0.26 to 0.36)	0.23	0.28	0.429	(−0.33 to 0.79)
Daily	−0.21	0.18	0.24	(−0.56 to 0.14)	0.11	0.09	0.263	(−0.29 to 0.08)	−0.03	0.08	0.702	(−0.19 to 0.13)	−0.001	0.09	0.988	(−0.19 to 0.18)	−0.31	0.17	0.071	(−0.64 to −0.03)
Age	**0**.**04**	**0**.**01**	**0**.**000**	(0.02–0.06)	0.007	**0**.**004**	0.114	(−0.002 to 0.02)	**0**.**009**	0.004	**0**.**021**	(0.001–0.017)	**0**.**009**	0.004	**0**.**039**	**(0.0004–0.02)**	**0**.**04**	0.008	**0**.**000**	**(0.02–0.06)**
BMI	−0.03	0.02	0.085	(−0.06 to 0.003)	−0.005	0.008	0.556	(−0.02 to 0.02)	−0.003	0.007	0.637	(−0.02 to 0.01)	0.003	0.08	0.746	(−0.01 to 0.02)	−0.02	0.01	0.162	(−0.05 to 0.008)
Years in occupation	−0.004	0.01	0.71	(−0.02 to 0.02)	0.002	0.006	0.694	(−0.009 to 0.14)	−0.004	0.005	0.440	(−0.014 to 0.006)	−0.002	0.006	0.73	(−0.01 to 0.01)	−0.004	0.01	0.735	(−0.02 to 0.02)
Seafood intake past week	**0**.**66**	**0**.**23**	**0**.**01**	**(0.21–1.12)**	**0**.**07**	**0**.**11**	**0**.**553**	**(−0.16** to **0.30)**	**0**.**0002**	**0**.**1**	**0**.**998**	**(−0.20** to **0.20)**	**0**.**1**	**0**.**11**	**0**.**372**	**(−0.12** to **0.33)**	**0**.**64**	**0**.**21**	**0**.**002**	**(0.23–1.05)**
PM exposure home (hours last week)	0.003	0.005	0.48	(−0.006 to 0.013)	0.004	0.002	0.070	(−0.0004 to 0.009)	−0.001	0.002	0.774	(−0.005 to 0.004)	−0.003	0.002	0.282	(−0.007 to 0.002)	0.007	0.004	0.089	(−0.001 to 0.02)
PM exposure work (hours last week)	−0.08	0.05	0.13	(−0.18 to 0.02)	0.022	0.03	0.398	(−0.03 to 0.07)	0.03	0.023	0.234	(−0.02 to 0.07)	−0.017	0.03	0.501	(−0.07 to 0.34)	**−0**.**10**	0.05	**0**.**039**	**(−0.19** to **−0.005)**
Blood selenium	**0**.**02**	**0**.**004**	**0**.**000**	**(0.008–0.03)**	0.003	**0**.**002**	0.119	(−0.001 to 0.008)	0.0009	0.002	0.623	(−0.003 to 0.004)	−0.0003	0.002	0.868	(−0.005 to 0.004)	**0**.**02**	0.004	**0**.**000**	**(0.009–0.02)**
Welding	−0.32	0.35	0.36	(−1.01 to 0.37)	0.06	0.17	0.742	(−0.29 to 0.40)	0.11	0.15	0.489	(−0.20 to 0.41)	0.23	0.18	0.183	(−0.11 to 0.58)	−0.23	0.32	0.467	(−0.86 to 0.40)
Shootingrange													0.32	0.33	0.348	(−0.35 to 0.98)				

Values in bold are statistically significant.

We only found statistically significantly lower concentrations of blood chromium in the smelter worker group compared to the office-worker group after adjustment for the factors from Table 1. Our findings of lower chromium in smelter workers were statistically robust with a delta *R*^2^ of 6.01%, above the threshold of 5.03% (Supplementary Materials). In all the other metals, we did not find statistically significant differences and sensitivity analysis showed that these findings were underpowered, and a type II error may be present.

We found several statistically significant predictors within the heavy metals. The predictors that were statistically significant with delta *R*^2^ was higher arsenic concentrations with increasing age (*P* < 0.001), higher blood selenium (*P* = 0.001), and participants with seafood intake last week (*P* = 0.004). In cadmium, sensitivity analysis showed the effect to be statistically robust in sometimes and daily smokers. In mercury, we found higher concentrations of mercury in the blood samples of those with seafood intake last week (coeff: 0.63, *P* = 0.002), older aged participants (coeff: 0.04, *P* < 0.001), and those with higher blood selenium concentrations. These findings were statistically robust. The statistically significant factors with delta *R*^2^ below 5.03% are discussed in the Supplementary Materials.

### Cross-week changes of heavy metals

Overall, we detected no statistically significant increase of the heavy metals from Day 1 to Day 4 for the PM-exposed groups using Wilcoxon's signed-rank test (Table 4). Instead, we found a statistically significant cross-week decrease in blood lead concentrations in chimney sweepers, and an increase in the unexposed group. The median change for the groups lead was −4.91% and 2.91%, respectively, within the analytical variation (vKa) for both at 5%.

**Table 4:** Wilcoxon's matched pair signed-rank test cross-week difference.

	Median			
Metal/occupation	Day 1 µg/L	Day 4 µg/L	*Z*-score	*P*-value^a^
**Arsenic**				
Smelterworker	0.98	1.06	1.34	0.179
Chimney sweeper	0.88	0.99	0.12	0.908
Unexposed	2.79	2.74	1.86	0.064
**Cadmium**				
Smelterworker	0.27	0.22	1.381	0.134
Chimney sweeper	0.25	0.22	1.464	0.145
Unexposed	0.24	0.26	0.373	0.714
**Chromium**				
Smelterworker	0.38	0.34	1.05	0.300
Chimney sweeper	0.51	0.50	1.320	0.189
Unexposed	0.43	0.51	−1.879	0.606
**Lead**				
Smelterworker	8.59	8.76	−0.169	0.872
Chimney sweeper	9.35	8.90	2.340	0.019
Unexposed	9.53	9.80	−1.984	0.047
**Mercury**				
Smelterworker	0.61	0.61	0.006	1.000
Chimney sweeper	0.61	0.56	0.288	0.776
Unexposed	1.35	1.27	0.193	0.851

^a^Wilcoxon's signed-rank test, exact probability.

## Discussion

Studies indicate that ferro-silicon smelter workers and chimney sweepers are exposed to heavy metals through particulate matter in their work environment (Bagchi and Zimmerman 1980; Andersson 1987; IARC Working Group on the Evaluation of Carcinogenic Risks to Humans 2012; Næss et al. 2014; Kero et al. 2017; Berglund 2018). Our primary hypothesis was that smelter workers and chimney sweepers have higher blood concentrations of whole blood heavy metals than office workers, and that an increase of the metals during a 4-d workweek could be detected, despite the use of properly fitted RPE.

In general, the blood concentrations of all metals in our participants were under the reference concentrations set by The Mayo Clinic for most participants (Table 2) (Mayo Clinic Laboratories). Contradictory to our hypothesis, smelter workers and chimney sweepers did not have higher heavy metal concentrations compared to office workers after adjustment for the relevant factors in Table 1. We believe their high use of RPE (>95% of the time) might be the main reason. Surprisingly, we found statistically significantly lower chromium concentrations in smelter workers, but individual predictor analysis did not indicate a reason. We speculate that this might be due to lower dietary chromium intake.

### Smelter workers

Analyzing iron and silica would have been more relevant in the exposure assessment in addition to the selected heavy metals. However, the impact of silica and iron are difficult to interpret in blood samples due to the heavy influence of dietary intake and more. In a study from a ferro-silicon plant in Egypt, cadmium, manganese, iron, nickel, and lead in the raw materials and dust and fumes within the factory were measured (Soltan et al. 2005). They found mostly manganese, iron, nickel, and cobalt in their samples from the air, while cadmium and lead were depleted. A Norwegian study, which sampled the furnace fumes in a ferro-manganese smelter plant, found high concentrations of arsenic, beryllium, lead, copper, chromium, nickel, and vanadium in the fumes inside the furnace (Næss et al. 2014). This study did not measure actual heavy metal content in the airborne dust in the furnace hall. It was assumed that the content of these heavy metals in the work atmosphere would be low (Kero et al. 2017). All these previously published findings indicate that the concentrations of the heavy metals we chose to measure may be low in the ambient work atmosphere of ferro silicon smelter workers. In addition, smelter workers reported over 95% use of RPE in exposed areas. This would further reduce the risk of heavy metal uptake, as the primary route of uptake of heavy metals is through the airways. Skin uptake of heavy metal has been studied in Sweden in a hard metal plant producing cobalt (Andersson et al. 2024). They found that cobalt on skin was correlated significantly to cobalt in blood, which indicates that heavy metal uptake through skin is plausible. But we could not replicate this in our study. Therefore, the observed low blood concentrations in the particle-exposed individuals in this study support the assumption of low ambient presence of heavy metals in the work atmospheres together with their use of RPE.

### Chimney sweepers

Few studies have investigated heavy metal exposure in chimney sweepers. In Sweden, studies found the heavy metal content in soot may only be up to 5% of their limit value in air (Andersson 1987; Berglund 2018), meaning the exposure is extremely low. Together with their exceptional use of RPE, this study supports our findings that the blood concentrations, and therefore possibly the exposure, of heavy metals in chimney sweepers may be low.

### Cross-week sampling

We found statistically significant decreasing cross-week concentrations of lead in chimney sweepers and increasing concentrations of lead in the unexposed group, though within the analytical variation (vKa) of 5%, meaning we could not determine that these statistical differences reflect an actual change in blood lead concentration.

We speculate on several reasons where the PM-exposed workers may be properly protected against exposure through RPE use, the uptake through skin may be low, exposure is not reflected in the full blood samples, or the rate of uptake may be poorly coordinated with our chosen sampling period because of our chosen heavy metal half-lives. In addition, low presence of the investigated heavy metals in the workers’ atmosphere may be a factor. The points are discussed for each occupation separately below. Based on half-lives of our chosen heavy metals, different sampling intervals would have been more appropriate. For instance, sampling of arsenic should have been conducted on the same day, based on its short half-life in blood of 4 to 6 h for inorganic and 10 to 20 h for the organic arsenic (Martinez-Morata et al. 2023). For blood lead, a more optimal interval could have been 1 to 3 mo, and their cumulative blood lead index could have been calculated, which may have produced more accurate results (Hu et al. 2007). However, a study like this has not been performed previously in these occupations, and we wanted to include as many heavy metals as possible, together with other chemicals not included in this article and keep the burden on participants as low as possible.

### General considerations

We have not found studies with similar occupational data in the published literature; thus, our results are novel and cannot be compared to similar other studies. Compared to 2 population-based studies from Norway, our results showed low blood concentrations of heavy metals (Averina et al. 2020; Syversen et al. 2021). The geometric means of arsenic, cadmium, chromium, lead, and mercury in the blood samples in our study were 88%, 16%, 41%, 85%, and 380% lower, respectively (Table 2) compared to what was found in HUNT3 (21, 22). Compared to the Tromsø blood donor study, our participants’ arithmetic means of Cd, Pb, and Hg were 26%, 35%, and 97% lower, respectively (Table 2) (Averina et al. 2020). Temporal changes in exposure from the surroundings may explain some of these differences in blood levels of metals, but also year of sampling. Blood samples analyzed in HUNT3 were, for example, collected between 2006 and 2008, whereas the samples in the Tromsø blood donor study were collected in 2015 to 2016. Nonetheless, individual predictors were consistent with findings in this study, with higher heavy metals in those with seafood intake, smoking, and higher age.

### Limitations

A key limitation of the study relates to the statistical power for detecting small individual predictor effects. The sensitivity analysis proved that the sample size was sufficient to detect a unique predictor contribution (delta *R*^2^) of 5.03% or greater with power of 80%. Consequently, any predictor that did not reach statistical significance and had a calculated delta *R*^2^ below this threshold must be interpreted cautiously (Supplementary Materials). Their nonsignificant findings may not reflect the true absence of an association, but rather the study's insufficient statistical power to reliably detect a genuinely small effect size. A larger total number of participants (calculated to *N* = 310) would have reduced the risk of type II error. We therefore cannot conclude that there is no difference between the office-worker group and the exposed occupations in these metals, only that our study did not have enough power to detect the findings.

We did not perform personal air sampling of heavy metals due to logistical reasons and lack of funding, which could have given us detailed information on the workers’ heavy metal exposure through air and particulate matter. We do not have detailed information on other types of relevant exposure, such as dental amalgam, orthopedic prothesis, alcohol consumption, medications that may contain heavy metals, levels of heavy metals in water, etc.

Because of the complexity of biological monitoring of heavy metals, measurement of certain heavy metals in whole blood requires speciation of metabolites to aid assessment of toxicity (Martinez-Morata et al. 2023). In arsenic and mercury, this is required, since seafood consumption exposes the body to organic mercury and arsenic, making biological monitoring and interpretation difficult. Whole blood samples analyzed in our study reflect the total amount of the heavy metal and do not differentiate on the heavy metal state and form. Thus, we do not know if our participants’ blood consists of the toxic metals or not. The cost of speciation analysis would exceed budget. Despite we have compared our findings to other population studies (Simić et al. 2022; Syversen et al. 2021). These have not differentiated the metal species either.

## Conclusion and practical implications

We found low blood concentrations of heavy metals in our study population, lower than what has previously been found in Norwegian population-based studies. We found no differences between PM-exposed occupations and office workers after adjusting for the factors and we found no detectable increase in cross-week blood concentrations of heavy metals in smelter workers or chimney sweepers. Analyses suggested that the main factors influencing the metal blood concentration were previously known factors, such as tobacco smoking, age, and seafood intake, indicating the environmental exposures outside work were of greater significance than heavy metal pollution in the ambient air at work. This implicates that their use of RPE is important, and preventive measures in lifestyle measures such as smoking would further improve their heavy metal load.

## Supplementary Material

wxaf089_Supplementary_Data

## Data Availability

Our data consists of information that could potentially lead to backward identification of individuals, and individual privacy would be compromised if the data were made available.
