# Supplementary material for: Heavy metal blood concentrations in smelters and chimney sweepers in Norway
Source: Ann Work Expo Health. 2026 Jan 21;70(1):wxaf089. doi: 10.1093/annweh/wxaf089 (PMC12822786; doi:10.1093/annweh/wxaf089)
Supplement: wxaf089_Supplementary_Data [file wxaf089_supplementary_data.pdf]

Supplementary materials

Heavy metal blood concentrations in smelters and chimney sweepers in Norway.

Teigen, Krister Aune[1,2*], Höper, Anje Christina [1,2], Huber, Sandra[3], Hegseth, Marit Nøst [1,2].

[1] Department of Occupational and Environmental Medicine, University Hospital of North Norway, Tromsø, Norway.
[2] Department of Community Medicine, UiT the Arctic University of Norway, Tromsø, Norway.
[3] Department of Laboratory Medicine, University Hospital of North Norway, Tromsø, Norway.

The table below shows the results of the sensitivity power analysis.

We used the F-test for linear multiple regression: Fixed model, R^2 deviation from zero with the observed sample size (N=151), the number of predictors (k=25, 26 in lead), and an alpha error probability of 0.05 with power=0.8. The resulting threshold effect size (delta R^2) in the model was calculated to be 17.4% and 17.7% in lead. For each predictor, the threshold effect size (delta R^2) was calculated to 5.03% and 5.00% in lead. For simplicity we used 5.03% and 17.7%. The delta R^2 was used to interpret the power of the unique contribution of each predictor with the power of 80%.

Regression model:

Arsenic, cadmium, chromium and mercury:

F tests - Linear multiple regression: Fixed model, R^2 deviation from zero
Analysis: Sensitivity: Compute required effect size
Input: α err prob = 0.05
Power (1-β err prob) = 0.80
Total sample size = 151
Number of predictors = 25
Output:
Noncentrality parameter λ = 26.2890639
Critical F = 1.5942806
Numerator df = 25
Denominator df = 125
Effect size f^2 = 0.1740998

Lead:

F tests - Linear multiple regression: Fixed model, R^2 deviation from zero
Analysis: Sensitivity: Compute required effect size
Input: α err prob = 0.05
Power (1-β err prob) = 0.80
Total sample size = 151
Number of predictors = 26
Output:
Noncentrality parameter λ = 26.8350429
Critical F = 1.5851561
Numerator df = 26
Denominator df = 124
Effect size f^2 = 0.1777155

Each predictor:

F tests - Linear multiple regression: Fixed model, R^2 deviation from zero
Analysis: Sensitivity: Compute required effect size
Input: α err prob = 0.05
Power (1-β err prob) = 0.80
Total sample size = 151
Number of predictors = 1
Output:
Noncentrality parameter λ = 7.9512763
Critical F = 3.9046281
Numerator df = 1
Denominator df = 149
Effect size f^2 = 0.0526575

Delta $R^2 = f^2 / (1+ f^2) = 0.053 / (1+0.053) = 0.0503 = 5.03\%$.

Calculation table:

Metal Model	Predictor	Beta-coeff	Std. Error	t-statistic (beta/SE)	Calculated delta R^2	Power Status
**Arsenic**	Blood selenium	0.02	0.004	5	**0.1667 (16.67%)**	Extremely Robust
	Age	0.04	0.01	4	**0.1135 (11.35%)**	Extremely Robust
	Seafood intake	0.66	0.23	2.87	**0.0618 (6.18%)**	Robust
	Daily Smokers	-0.68	0.3	-2.27	0.0399 (3.99%)	Marginal <5.03%
**Cadmium**	Daily Smoking	1.3	0.16	8.13	**0.3459 (34.59%)**	Extremely Robust
	Sometimes Smoking	0.64	0.13	4.92	**0.1622 (16.22%)**	Extremely Robust

	Previously Smoking	0.24	0.11	2.18	0.0366 (3.66%)	Marginal <5.03%
**Chromium**	Smelter workers	-0.45	0.16	-2.81	**0.0601 (6.01%)**	Robust
	Age	0.009	0.004	2.25	0.0389 (3.89%)	Marginal <5.03%
**Lead**	Age	0.009	0.004	2.25	0.0392 (3.92%)	Marginal <5.03%
	Sometimes Smoking	0.29	0.13	2.23	0.0385 (3.85%)	Marginal <5.03%
	Daily Smoking	0.32	0.16	2	0.0313 (3.13%)	Marginal <5.03%
	Previously Smoking	0.23	0.11	2.09	0.0340 (3.40%)	Marginal <5.03%
**Mercury**	Age	0.04	0.008	5	**0.1667 (16.67%)**	Extremely Robust
	Seafood intake	0.64	0.21	3.05	**0.0693 (6.93%)**	Robust

We only found statistically significantly lower concentrations of blood chromium in the smelter worker group compared to the office-worker group after adjustment for the factors from Table 1. Our findings of lower chromium in smelter workers were statistically robust with a delta R^2 of 6.01% (threshold 5.03%). Age was the only factor which had a statistically significant impact on the chromium concentrations (p=0.026), but the effect size was small (delta R^2 = 3.92%). In all the other metals, we did not find statistically significant differences and sensitivity analysis showed that the unique contribution of being a smelter worker or chimney sweeper only contributed to maximum 2.49% (delta R^2) in those metals. This means that these findings were underpowered, and a type II error may be present. A larger number of participants (calculated to N=310) would have reduced the risk of type II error. We therefore cannot conclude that there is no difference between the office worker group and the exposed occupations in these metals, only that our study did not have enough power to detect the findings.

Individual predictor analysis found several statistically significant factors. In arsenic we found statistically significant lower concentrations in daily smokers (p=0.023). The delta R^2 found this to be slightly below the threshold of 5.03%, meaning this finding may be sensitive to

error. Further, we found higher arsenic concentrations with increasing age ($p<0.001$) in those with higher blood selenium ($p=0.001$) and participants with seafood intake last week ($p=0.004$). These findings were statistically robust with delta R^2 over 5.03% (supplementary materials)

In cadmium we found statistically significant higher concentrations with increasing smoking status. The coefficients increased two-fold from “previous smokers” to “sometimes smokers” (coeff: 0.24 and 0.64 respectively), and from “sometimes smokers” to “daily smokers” (coeff: 0.64 and 1.30 respectively). Sensitivity analysis showed the effect to be statistically robust in sometimes and daily smokers (delta $R^2 > 5.03\%$).

For lead, we found that increasing smoking statuses had higher concentrations of lead. The daily smokers had the higher concentrations of lead in their blood (coeff: 0.31 $p=0.045$) compared to the never smokers (coeff: 0.23, $p=0.046$). But these findings were not statistically robust. Furthermore, older age was statistically significantly associated with higher concentrations of lead (coeff: 0.009, $p=0.05$). In addition, being a female was associated with lower concentrations of lead (coeff: -0.36, $p=0.004$). These findings were not statistically robust.

For mercury we found higher concentrations of mercury in the blood samples of those with seafood intake last week (coeff: 0.63, $p=0.002$), older aged participants (coeff:0.04, $p<0.001$) and those with higher blood selenium concentrations. These findings were statistically robust. Contrary to our hypothesis, there was lower blood concentrations of mercury in those with higher reported particulate matter exposed hours at work during our sampling period (coeff: -0.01, $p=0.039$), but this finding proved to be under powered with a delta R^2 under 5.03%.
